# P05.23. Emotional awareness through mindful body awareness training contributes to maintained abstinence among women in substance use disorder recovery

**DOI:** 10.1186/1472-6882-12-S1-P383

**Published:** 2012-06-12

**Authors:** C Price, K Smith-DiJulio

**Affiliations:** 1 University of Washington, Seattle, USA

## Purpose

Results of a recent National Insitute of Drug Abuse-funded pilot randomized controlled trial of Mindful Awareness in Body-oriented Therapy (MABT) for women in substance use disorder treatment showed improved substance use outcomes for MABT compared to treatment as usual. In addition 80% of the MABT participants maintained a daily mindful body awareness practice at nine month follow-up. The purpose of this subsequent study was to explore the perceived role of MABT and mindful body awareness practice in the recovery process.

## Methods

A qualitative design utilizing a focus group method was employed. Participants from the larger RCT that had completed the MABT intervention (n =18) were recruited for the focus group, and five attended. Semi-structured questions were used to explore participants’ utilization of mindful body awareness practice in substance use disorder recovery, and the usefulness of various aspects of MABT delivery. Content analysis was used. Two researchers individually coded for themes across participant responses and together verified the coded themes.

## Results

The primary themes were: (1) motivation to maintain body awareness practices one year post-intervention was due to improved ability to access and process emotions when using these practices; (2) exploration of uncomfortable feelings was perceived as critical for relapse prevention, as substance use was associated with escalated emotions and an inability to attend to feelings; (3) MABT facilitated awareness of inner experience and the connection between emotions and the body; they had not been able to achieve this awareness with prior therapeutic approaches. Also, three aspects of MABT were considered critical for learning mindful body awareness practice: (1) individual delivery to allow for safe exploration of emotional experiences, (2) manual/touch-based processes to learn interoceptive skills, and (3) homework to integrate body awareness practice into daily life.

## Conclusion

These findings point to the importance of studying emotional awareness in mind-body interventions, and in women’s substance use disorder prevention and treatment specifically.

